# Editorial: Neuroinflammation in acquired epilepsy

**DOI:** 10.3389/fcell.2022.1074537

**Published:** 2022-11-07

**Authors:** Jianxiong Jiang, Vijayalakshmi Santhakumar, Xinjian Zhu

**Affiliations:** ^1^ Department of Pharmaceutical Sciences, College of Pharmacy, The University of Tennessee Health Science Center, Memphis, TN, United States; ^2^ Department of Molecular, Cell and Systems Biology, University of California, Riverside, Riverside, CA, United States; ^3^ Department of Pharmacology, Medical School of Southeast University, Nanjing, China

**Keywords:** epileptogenesis, BDNF, biomarker, brain infection, NADPH oxidase, oxidative stress, reactive oxygen species

In spite of astonishing advances in epilepsy research and treatment over the past few decades, epilepsy remains one of the most common and devastating brain diseases and still affects approximately 65 million people globally (Devinsky et al., 2018). In addition to their wide-ranging side effects, antiseizure drugs (ASDs) are not effective in controlling seizures in more than 30% of patients who have pharmacoresistant epilepsy (Janmohamed et al., 2020). It is rather unfortunate that current medications provide merely symptomatic relief and have not been demonstrated to prevent epilepsy in people at risk or modify the disease progression (Galanopoulou et al., 2021). Therefore, there remains an urgent need for alternative antiepileptic treatments, despite the rapid expansion of modern ASDs that emerged during the first 2 decades of this century (Varvel et al., 2015; Löscher and Klein, 2020; Yu et al., 2022).

Mounting lines of evidence support an essential role for proinflammatory mediators in the brain in acquired epileptogenesis, a pathogenic process that is proposed to transform a normal brain to one generating seizures following various brain insults, such as *de novo* status epilepticus (SE), brain infections, traumatic brain injuries, brain tumors, and strokes (Yu et al., 2019; Korgaonkar et al., 2020; Terrone et al., 2020). It has been widely proposed that modulating key proinflammatory mediators might disrupt the epileptogenic processes and lead to modification and/or even prevention of epilepsy. In the current Research Topic, we bring together a diverse collection of primary research and review articles that highlight the roles of brain inflammation in epilepsy of various etiologies and pathogeneses.

Both inflammation and oxidative stress are well known for their pathophysiological roles in the epileptic brain. However, they are often studied as separate entities despite the evidence that the redox-based signaling cascades and inflammatory reactions have extensive crosstalk (Fabisiak and Patel). Recent studies have uncovered a variety of mechanisms whereby oxidative stress and neuroinflammation greatly influence each other in the context of epilepsy. For instance, neuroinflammation can be regulated by transcription factors such as NF-κB and nrf2 that are activated by reactive oxygen species (ROS). Neuroinflammation in turn can induce the expression and activity of NADPH oxidase (NOX), fostering a highly oxidative environment. Moreover, the oxidative and proinflammatory mediators can moulate distinct intracellular pathways expressed in different cell-types, exemplified by NOX-2 dependent increase in ROS in neurons and astrocytes triggered by SE, and myeloid differentiation primary response 88 (MyD88) dependent glial activation through Toll-like receptors (TLRs) (Almeida et al.). The reviews presented in this collection highlight how signaling crosstalk between neuroinflammation and oxidative stress and their cell type specific roles may be leveraged for novel therapeutic strategies for epilepsy.

Neuroinflammatory processes triggered by acute brain insults such as SE are highly regulated and show time- and age-dependency. Using a rat model of kainate-induced SE, Erisken et al. demonstrate prolonged induction of many key inflammatory genes, particularly those associated with stress-activated protein kinases, p38 and JNK signaling pathways, uniquely in adult brains. In contrast, many of the same genes show relatively transient expression in developing brains under similar experimental conditions, suggesting that the immature brains might be more resistant to SE-induced cell death and neuropathology. In line with findings in adult animals, hippocampal tissues from mesial temporal lobe epilepsy patients showed upregulation of inflammation-related genes. These results highlight the association between uncontrolled neuroinflammation and epileptogenesis and suggest that epileptic seizures might result from prolonged activation of neuroimmune processes beyond the homeostatic threshold.

Brain infection is a leading cause of epilepsy, but the underlying molecular mechanisms are poorly understood. Patel et al. use Theiler’s murine encephalomyelitis virus (TMEV) infection to generate acute brain inflammation and the subsequent spontaneous seizures in mice. They show TMEV infection-induced seizures likely due to impaired GABAergic inhibition, secondary to alterations in neuronal intracellular chloride regulation. Their results further suggest that the brain-derived neurotrophic factor (BDNF) might contribute to the development of brain infection-triggered seizures by reducing the expression of K^+^/Cl^−^ cotransporter 2 (KCC2). This has the potential to enhance accumulation of intracellular chloride and increases excitability by rendering GABA depolarizing instead of hyperpolarizing as observed in chemoconvulsant models of SE (Pathak et al., 2007; Yu et al., 2013). Notably, the upregulation of brain BDNF observed in TMEV-infected mice has also been found in chemoconvulsant models of SE (Zhu et al., 2012; Thomas et al., 20162016; Yu and Jiang 2020), and is believed to contribute to acquired epileptogenesis by acting on its high-affinity receptor, the tropomyosin related kinase B (TrkB) (Lin et al., 2020).

Exposure to diisopropylfluorophosphate (DFP), a structural analog of type G chemical warfare agents (e.g., sarin and soman), is well known to induce SE, gliosis, neuronal death, and eventually the development of spontaneous recurrent seizures in rodents. Gage et al. show that both male and female rats which experience DFP-induced SE develop unique regions of glial scarring in the piriform cortex and amygdala, but not in the hippocampus. DFP-induced cortical glial scars are characterized by a massive clustering of reactive microglia, with increase in Iba1-and CD68-positive cells, surrounded by hypertrophic astrocytes and a decrease in NeuN-positive neurons in the scar core. Although female rats have been shown to require a higher dose of DFP to induce SE when housed in a room with only females, Rao et al. demonstrate that when both sexes are housed in the same room and administered the same DFP solution, SE severity was not different between sexes. These results reinforce the importance of sex as a key biological variable in experimental design and suggest that housing animals of both sexes together and using the same batch of test reagents will reduce experimental variability.

The benefits of low-intensity physical exercise to the CNS have been shown in animal models and patients with neurological diseases, such as Alzheimer’s disease, Parkinson’s disease, stroke, epilepsy, multiple sclerosis, anxiety and depression (Allendorfer and Bamman, 2018). Jia et al. demonstrate that the conventional ASD, valproate, combined with low-intensity exercise can reduce seizures and associated co-morbidities in kainate-treated mice. The reduction in seizure burden appears to be correlated with the suppression of inflammatory cytokines (IL-1β, IL-6, and TNF-α) and the immune receptor TLR4 in the hippocampus. Given that TLR4 is involved in epileptogenesis of diverse etiologies (Maroso et al., 2010; Korgaonkar et al., 2020), these findings suggest that the non-pharmacological intervention like low-intensity exercise might reduce neuroinflammation and provide an adjunctive strategy to enhance efficacy of conventional ASDs to treat epilepsy.

Developing preventive treatment for epilepsy is challenging because it is currently impossible to identify individuals that will develop epilepsy after initial precipitating brain insults. Theoretically, biomarkers that identify “at risk” individuals would facilitate the development of potential antiepileptogenic treatment (Simonato et al., 2021). By reviewing data from 60 human patients with focal epilepsy of autoimmune etiology, Sakamoto et al. propose a diagnostic algorithm that might help to predict the underlying autoimmune etiology of epilepsy before antibody testing results become available. Over 30% of epilepsy patients suffer from pharmacoresistant seizures associated with cognitive and psychiatric co-morbidities. Analyzing a microarray dataset from the Gene Expression Omnibus database, Min et al. identify 25 genes differentially expressed in the peripheral blood of patients with valproate resistance in epilepsy and significantly enriched in T-cell receptor recognition. While the potential confound posed by the differential seizure burden between valproate sensitive and resistant groups needs to be considered, these findings suggest that the peripheral blood T-cells and the differentially expressed genes could serve as biomarkers for refractory epilepsy. Identification of reliable biomarkers for diverse types of epilepsy and pharmacoresistance could facilitate both early diagnosis and development of new therapies, needed to achieve the ultimate goals of “no seizures, no side effects, and no co-morbidities” in epilepsy treatment.

The series of articles presented here address diverse ways in which neuroinflammation could shape acquired epilepsy and offers insights into how these processes may be leveraged to inform mechanisms of epileptogenesis, identify biomarkers, and to develop novel strategies for disease modification and treatment in epilepsy.
